# Advances in treating HER2-positive breast cancer: an interview with Sunil Verma

**DOI:** 10.1186/s12916-014-0129-y

**Published:** 2014-08-12

**Authors:** Sunil Verma

**Affiliations:** Sunnybrook Health Sciences Centre, 2075 Bayview Ave., Room T2 045, Toronto, ONM4N 3M5 Canada

**Keywords:** HER2, Breast cancer, Oncology

## Abstract

In this podcast, we talk to Dr Sunil Verma about the evolution of HER2 as a therapeutic target in breast cancer, and about how targeted therapy has revolutionized breast cancer treatment. We also discuss new agents in clinical development and the ways in which advances in treating HER2-positive breast cancer can be used to inform treatment decisions in other areas of medicine.

The podcast for this interview is available at: http://media.biomedcentral.com/content/movies/supplementary/SunilVerma-QandA.mp3.

## Introduction

**Figure 1:**
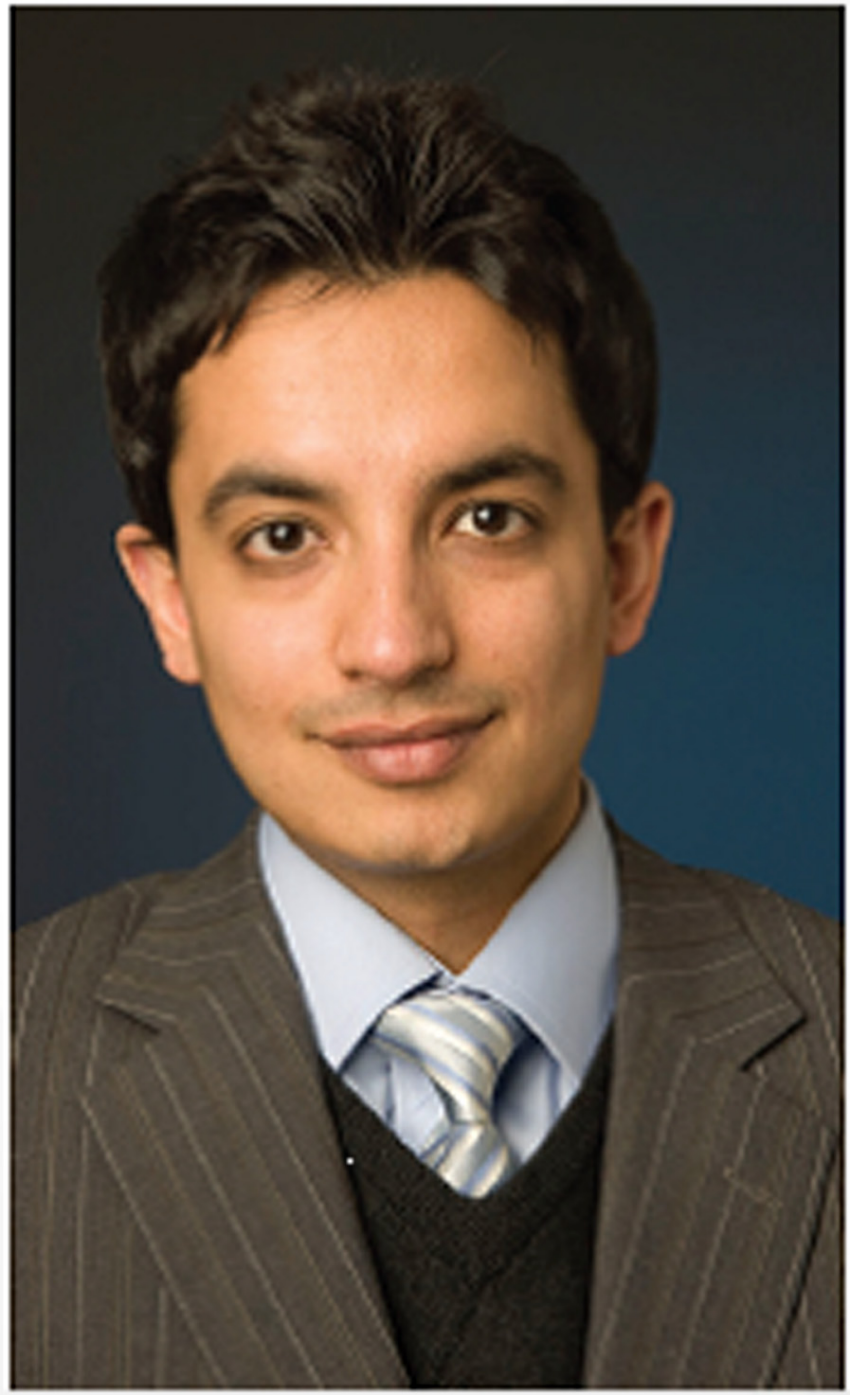
**Dr Sunil Verma.**

Dr Sunil Verma (Figure 1) is a medical oncologist who is internationally recognized for his research and education leadership in breast cancer. He is the principal investigator for many clinical trials in breast and lung cancer, and has led pioneering trials showing that the antibody-drug conjugate T-DM1 extends survival in women with advanced human epidermal growth factor receptor 2 (HER2)-positive breast cancer.

In this interview, we talk to Dr Verma about how HER2 has evolved into one of the most promising targets in breast cancer therapy, about new therapies and combinations that are under clinical development, and how insights gained into HER2-positive breast cancer can inform treatment decisions across oncology.

The podcast for this interview is available at: http://media.biomedcentral.com/content/movies/supplementary/SunilVerma-QandA.mp3.

### 1. Could you tell me a bit about yourself and how you got into working in the field of breast cancer?

I trained at the University of Alberta, which is in Edmonton, Canada. Early on in my career I was quite amazed by the fight, the bravery, and the courage of cancer patients. I thought, ‘what an incredible way to battle this disease’. I, in many ways, wanted to be part of that battle with them, and to be able to learn from how these patients live their lives and how they are able to manage such a life-transforming experience. Being part of that journey was quite empowering for them and also for the healthcare workers as well. So that’s really what attracted me towards oncology.

Through training in oncology, I met some wonderful mentors along the way. One in particular is Dr John Mackey, who is based at the Cross Cancer Institute in Edmonton. Seeing how he worked and his expertise in breast cancer is really what excited me to join the field. From there on, I did a fellowship in breast cancer at Sunnybrook Cancer Center in Toronto, and came on staff here about 10 years ago.

I now lead the breast cancer trials group here at Sunnybrook. The research that we do is across the spectrum – from quality of life to assessing and caring for end of life patients. However, our main focus, and my main focus, is in the HER2-positive arena. Of course I partake in research across the board; we have a number of fellows and residents who work with us, and it is a really great opportunity to conduct research and also teach others how to do research in breast cancer.

### 2. Thirty years after its discovery, HER2 is one of the most promising targets in breast cancer therapy. Could you take us through how HER2-directed treatments have developed and how they have impacted therapy, particularly with early breast cancer?

It is a journey 30 years in the making. The HER2 gene was identified around 1984, and this was followed with work by pivotal researchers, including Dennis Slamon, indicating that breast cancer patients who have HER2 overexpression and amplification had the worst prognosis. The advent of trastuzumab occurred in the early 1990s, and in the late 1990s it was shown that the incorporation of trastuzumab with standard chemotherapy improves overall survival. By the late 1990s, we had this information, and by 2000 we were using it for treatment of metastatic advanced HER2-positive breast cancer.

Over the past decade, I think our understanding of HER2-positive breast cancer has transformed significantly, going from a sense of using a targeted drug with chemotherapy – in effect, you lose the essence of targeted treatment when you’re giving it along with chemotherapy – to a point where we’re now using dual targeted therapy with trastuzumab and lapatinib, and dual targeted therapy with two antibodies (trastuzumab and pertuzumab). Our own work has highlighted the role of antibody-drug conjugates, moving away from traditional chemotherapy. I say this is truly a ‘targeted treatment’, as it incorporates both targeted therapy and targeted chemotherapy, in order to specifically target the HER2-positive cancer cells.

### 3. As well as their positive impact on early breast cancer, HER2-targeted therapies have shown promise for treating advanced breast cancer. For example, your clinical trials showed that an antibody-drug conjugate against HER2 prolonged survival in women with metastatic disease. Could you tell me a bit more about the impact of HER2-directed therapies on advanced breast cancer?

The use of trastuzumab in the adjuvant setting has improved overall survival, decreased the risk of recurrence by about 50% in relative terms, and has transformed our approach to treating breast cancer. However, we still have some patients who develop metastatic breast cancer, either *de novo* or patients who have had prior therapy and have relapsed. So, we still need to work a lot in understanding and treating our patients with advanced HER2-positive breast cancer.

In the past decade, we have gone from overall survival in the range of about 25 to 30 months with chemotherapy plus trastuzumab, to overall survival of more than 40 to 45 months with chemotherapy plus trastuzumab and pertuzumab in the first line setting. So, there has been a significant improvement in the first line setting. We have also learned that continued blockade of the HER2 receptor, either using antibodies or tyrosine kinase inhibitors (TKIs), is important to use alongside of chemotherapy and trastuzumab in the second line and beyond setting. The importance of chemotherapy and lapatinib has also been demonstrated in the second line and beyond setting, for patients who have progressed on trastuzumab.

I think one of the most significant advances in treating HER2-positive advanced breast cancer has been the use of antibody-drug conjugates. The concept of antibody-drug conjugates has been around for more than 30 years, but our study looking at trastuzumab emtansine, or T-DM1, was the first trial in solid tumors to show that such a concept can be harnessed to treat breast cancer patients. Not only does T-DM1 extend survival, but it also comes with less toxicity and fewer side effects compared with standard treatment. In oncology, it is rare to see treatments that lead to better outcomes and come with less toxicity. That is why the study was so pivotal. Now, T-DM1 would be considered standard care for patients who have progressed after first line anti-HER2 therapy.

### 4. How are HER2-directed agents being evaluated for early and advanced breast cancer?

The next focus is going to be looking at whether we can completely forgo chemotherapy in HER2-positive breast cancer. In our own research, we will investigate whether we can get the true essence of total targeted treatments through combining targeted therapies, whether it is trastuzumab-pertuzumab, trastuzumab-lapatinib, or other anti-HER2 partners. We will also be looking at the use of antibody-drug conjugates, either alone or in combination with other targeted drugs, and trying to see whether such an approach can be used in advanced cancer to delay the use of chemotherapy. Additionally, just as importantly, I think such an approach can be used in early breast cancer, where we can only reserve chemotherapy for a selected number of patients and use a total targeted treatment for most of our patients.

### 5. Which HER2-directed therapies are currently under clinical development?

There are a number of agents undergoing further evaluation. The three most used agents, of course, we have talked about; above and beyond trastuzumab, there is pertuzumab, lapatinib and T-DM1, which are in clinical use in most places around the world now. With respect to what is coming next, there have been some data suggesting that everolimus, an MTOR inhibitor, appears to be very promising in patients in the second line and beyond setting. However, in combination with chemotherapy, it was associated with significant toxicity. Everolimus may be of specific interest in patients who are hormone receptor-negative HER2-positive; I think the use of everolimus in this setting requires further evaluation.

There is significant interest in developing the second-generation tyrosine kinase inhibitors. One of the drugs is neratinib, which is currently being studied in a large Phase III trial called the NALA study, comparing neratinib and capecitabine versus capecitabine and lapatinib. Neratinib is a pan-HER inhibitor, a more potent tyrosine kinase inhibitor. Initially, there were some concerns with toxicity with diarrhea, but some of those concerns have been addressed by proper incorporation of anti-diarrheal medication with breast cancer treatment.

The next question is whether we can expand and build on this concept of combining chemotherapy and targeted therapy. One of the drugs that looks promising is called MM-302, which is a combination of Her-2 targeted antibody and anthracyclines. MM-302 is going to be going through Phase II and Phase III clinical trials shortly.

I think this area, by no means, has been fully understood. We need to continue to work on developing better therapies, and using the treatments that we have in a more user-friendly, patient-friendly, and patient-focused manner, to really harness the true strength and effectiveness of these treatments.

### 6. You have described some really promising results looking at different combinations of targeted therapies. Do you anticipate that these combinations will be used more routinely in the clinic in the future?

Yes, I think they will be. I think the best way to evaluate routine use is going to be increased participation and incorporation in the neoadjuvant setting. I think the more we can study these drugs in the neoadjuvant setting, where we have tumors evaluated before the start of treatment and after the completion of treatment, the better we can understand what is happening at the patient level. This will then help us to better understand the biology of tumors, the biology of how these drugs work, and the biology of resistance. I think this is where we are moving forward.

We also have a very unique opportunity to incorporate functional imaging along with assessment of pathology. Also, I think it would be wonderful to be able to assess functional diagnostics, in order to assess how these drugs are working through imaging.

Moving forwards, another goal is to be able to identify patients who are more likely to benefit from a total targeted treatment approach. The only way we could do that is by understanding the biomarkers of the tumor. If we can come up with a biomarker signature that tells us that if you have a total targeted treatment approach, your likelihood of clinical response is close to 100%, or your likelihood of a pathological complete response is above 90%, then we can start asking the question of whether we can potentially forgo surgery or radiation treatment for some of these patients when we are having such a significant benefit from targeted therapy.

I think we are just on the cusp of truly understanding how to use these targeted treatments. The only way we are going to be successful is not only by improving patient outcomes, but also seeing whether we can use these total targeted treatments in such a way that we can forgo some of the traditional weapons such as chemotherapy, radiation and surgery. I think that would be a significant achievement in not only breast cancer but oncology as a whole.

### 7. Could you tell me what your visions are for the future of treating HER2-positive breast cancer?

My vision, exactly as we were discussing, is to be able to offer a total targeted approach in the early breast cancer setting, forgoing chemotherapy for most patients, and only reserving chemotherapy for a selected few. The second step would be using targeted treatments where we can have a very good assessment of which patients are most likely to benefit. This would be a more selected, more cost-effective approach, and could have such a significant impact that we could forgo not only chemotherapy, but maybe surgery and radiation for some of our patients. In the advanced stage setting, my vision for the future is using tailored treatment based on specific biomarkers, where we tend to use a targeted approach initially, and use chemotherapy only at a later stage.

This, in a nutshell, summarizes the vision. In order for this vision to come true, I think we really need everybody on board, including industry, patient groups, patient advocacy organizations, researchers and scientists, to see how we can work together and how we can design trials to look at these very important questions. This should allow us to really shift the care to a chemotherapy-free approach and really improve the outcome of our patients and, at the same time, not introduce any significant side effects.

### 8. You have outlined some tremendous progress that has been made in treating HER2-positive breast cancer. Could you now tell me about other targeted approaches in solid tumors and how the results from clinical trials in HER2-positive breast cancer can inform treatment decisions in other cancer types?

The insights that we have gained from HER2-positive breast cancer have been tremendous. One of the first publications on combining chemotherapy and targeted treatments was in 2001, with the use of trastuzumab and chemotherapy. Then, we have the data for capecitabine and lapatinib, one of the first times we have shown that TKIs can be combined with chemotherapy. We also showed that TKIs can be used in combination with an antibody, with lapatinib and trastuzumab; this was one of the first times in oncology that such an approach was found to be so successful. The dual antibody approach with pertuzumab and trastuzumab was demonstrated by José Baselga in 2011; this was one of the first insights that we can combine two antibodies to gain synergy by targeting the same receptor. The antibody-drug conjugate concept that we published in 2012 showed that there is a significant achievement in efficacy without the side effects. Again, this was one of the first times in oncology that such an effect was shown.

These principles of combining antibodies, combining antibodies with TKI and the antibody-drug conjugate in HER2-positive breast cancer have taught us a lot of insights that are now applicable to many other tumor types. There is now a significant interest in combining two antibodies in colorectal cancer; the development of antibody-drug conjugates is now occurring across the board in many different tumor sites. The concept of combining an antibody and a TKI has gained significant interest in lung cancer research, looking at combinations of drugs like cetuximab and afatinib, showing that there is some synergy in preliminary studies. I think the insights that we have gained in how to target oncogenes in HER2-positive breast cancer are going to be applied across the board in many other tumor sites.

I think the model of care and the design of studies that we are currently using will have a significant reach above and beyond HER2-positive breast cancer. I think this journey has just started with the identification of targeted drugs and user-targeted drugs, but it still needs to be established how best to optimize their use and really harness their strength; I think this will have a significant impact above and beyond breast cancer.

### 9. Where can I find out more?

See reference list [1–6].
